# Futterverweigerung und anorektale Abszesse bei Mastschweinen möglicherweise verursacht durch eine Kontamination von Corn-Cob-Mix mit Gewöhnlichem Stechapfel ( *Datura stramonium* )

**DOI:** 10.1055/a-2730-9629

**Published:** 2025-11-27

**Authors:** Anton Schulte zu Sundern, Florian Lohkamp, Sabine Aboling, Christian Visscher

**Affiliations:** 1 Tierarztpraxis Lastrup-Löningen, Lastrup; 2 Institut für Tierernährung, Stiftung Tierärztliche Hochschule Hannover

**Keywords:** Nachtschattengewächse, Phytotoxine, Tropanalkaloide, Umfangsvermehrung, Futtermittelkontamination, Solanaceous plants, phytotoxins, tropane alkaloids, swelling, feed contamination

## Abstract

Im Oktober 2022 kam es in einem nordwestdeutschen Schweinemastbetrieb innerhalb weniger Tage zum vermehrten Auftreten von anorektalen Abszessen bei Endmastschweinen. Der Mäster hält ca. 3000 Schweine. Betroffen waren ausschließlich Endmastschweine (ca. 100kg) in verschiedenen Stallabteilen und -buchten. Die betroffenen Schweine wiesen eitrig-entzündliche Veränderungen zwischen Rektum, Anus und Schwanzansatz auf. Anzeichen mechanischer Verletzungen oder Kannibalismus konnten nicht nachgewiesen werden. Der Halter berichtete von einer plötzlichen auftretenden Futterverweigerung ca. 2 Wochen vor dem Auftreten der Abszesse. Ab diesem Zeitpunkt erfolgte der Einsatz einer neuen Charge Corn-Cob-Mix. Die Tiere erhielten Flüssigfutter aus Getreide (Gerste, Weizen), Corn-Cob-Mix und ein Ergänzungsfuttermittel. Im Anmischbehälter der Flüssigfütterung konnten untypische, schwarze, stecknadelkopfgroße Partikel festgestellt werden. Nach Rücksprache mit dem Landwirt, der den Mais für den Corn-Cob-Mix angebaut hatte, konnte Gewöhnlicher Stechapfel ( *Datura stramonium* ) als Kontamination ermittelt werden. Eine Anfälligkeit von Schweinen auf die antinutritiven Effekte von Stechapfel ist bekannt. Diese umfasst neben der Futterverweigerung auch eine Reduktion der Magen-Darmmotorik. Im vorliegenden Fall konnte vom Tierhalter eine Verstopfung bei betroffenen Schweinen nicht direkt beobachtet werden, scheint aber dennoch wahrscheinlich. Diese könnten in der Folge zu den beschriebenen anorektalen Abszessen geführt haben.

## Abstract (en)

In October 2022, an increased incidence of anorectal abscesses occurred within a few days in finishing pigs on a pig fattening farm in north-western Germany. The farm keeps approx. 3000 pigs. Only finishing pigs (approx. 100 kg) in various stall compartments and pens were affected. The affected pigs developed purulent-inflammatory changes in the region between rectum, anus, and base of the tail. Signs of mechanical injuries or cannibalism were not evident. The owner reported a sudden refusal to feed about two weeks prior to the appearance of the abscesses. A new batch of corn-cob-mix was used from this point onwards. The animals were given liquid feed consisting of grain (barley, wheat), corn-cob-mix and supplementary feed. Atypical black particles the size of a pinhead were found in the mixing container of the liquid feed. After consultation with the farmer who had grown the maize used for the corn-cob mix, common datura ( *Datura stramonium* ) was identified as a contaminant. Pigs are known to be susceptible to the antinutritive effects of datura. In addition to feed refusal, this also induces a reduction of gastrointestinal peristaltic movement. In the present case, constipation was not directly observed in the affected pigs by the farmer, however, this nevertheless appears to represent the likely cause of the dermal changes. Subsequently, the datura contamination could be involved in the pathogenesis of the described anorectal abscesses.

## Einleitung

Gewöhnlicher Stechapfel ( *Datura stramonium* ) aus der Familie der Nachtschattengewächse ist eine ursprünglich aus Amerika und Osteuropa stammende Pflanze. Inzwischen ist sie flächendeckend in Europa verbreitet und immer häufiger wildwachsend auf heimischen Äckern zu finden. Die Pflanze wächst aufrecht bis buschig und erreicht dabei 1,5 m Höhe. Markant sind sowohl ihre Blüten, die von Juni bis Oktober ausgebildet werden, als auch ihre stacheligen, namensgebenden Samenkapseln. Bevorzugte Verbreitungsgebiete sind für die als wärmeliebend geltende Pflanze sonnenexponierte Ruderal- und Unkrautgesellschaften.

Alle Pflanzenteile enthalten in unterschiedlichen Konzentrationen die Tropanalkaloide Atropin, Hyoscyamin und Scopolamin 1 2 . In niedrigen Dosen wirken diese als Acetylcholin-Antagonisten und heben so die Effekte des Parasympathikus auf. In toxischen Dosen regen sie das zentrale Nervensystem an 3 . Früher in der Humanmedizin als Arzneimittel bei Asthma und anderen Atemwegserkrankungen genutzt, werden stechapfelhaltige Präparate heutzutage hauptsächlich in der Alternativmedizin eingesetzt 4 5 .

Neben lebensbedrohlichen Stechapfel-Vergiftungen, die durch den Konsum als halluzinogene Arzneidroge herbeigeführt werden, kommt es sowohl in der Human- als auch Veterinärmedizin immer wieder durch kontaminierte Lebens- bzw. Futtermittel zu Vergiftungsfällen 6 7 8 .

## Fallbericht

### Anamnese und klinisches Bild

Im Oktober 2022 kam es in einem nordwestdeutschen Schweinemastbetrieb zu einem plötzlichen und deutlichen Rückgang der Futteraufnahme. Das Allgemeinbefinden der Tiere war nicht gestört und klinische Symptome fehlten. Im weiteren Verlauf fielen dem Halter kleine stecknadelkopfgroße Partikel im Anmischbehälter der Flüssigfütterung auf. Diese schwammen teilweise auf der flüssigen Phase des Flüssigfutters. Die Futterverweigerung der Schweine trat unmittelbar nach dem Wechsel des Corn-Cob-Mix (CCM) ein. Die Tiere erhielten Flüssigfutter aus Gerste, Weizen, Corn-Cob-Mix und ein Ergänzungsfuttermittel. Der hierfür verwendete Mais stammte aus der Ernte 2022 und wurde aus der Region beim selben Landwirt zugekauft. Andere Mäster wurden damit nicht beliefert. Eine Inspektion des Futters im Institut für Tierernährung der Stiftung Tierärztliche Hochschule Hannover ergab eine Kontamination mit Samen bzw. Teilen von Früchten des Gewöhnlichen Stechapfels ( *Datura stramonium* ), wahrscheinlich als Folge der Ernte von stark verkrautetem Mais. Abgesehen von diesen etwa stecknadelkopfgroßen Partikeln ließen sich durch die Sinnenprüfung keine produktuntypischen Abweichungen feststellen. Dies galt auch für den Geruch, der wie für Corn-Cob-Mix üblich einen leicht säuerlichen Charakter aufwies. In der Folge wurde der zugekaufte CCM weiter gefüttert, allerdings mit CCM anderer Herkünfte gestreckt und zusätzlich mit appetitanregenden Futtermittelzusatzstoffen (Aromastoffe) versetzt. Futterverweigerungen konnten daraufhin nicht weiter festgestellt werden und die Futteraufnahme entsprach dem Niveau vor dem Einsatz des zugekauften CCM. Das Allgemeinbefinden der Mastschweine war weiterhin ungestört.

**Abb. 1:**
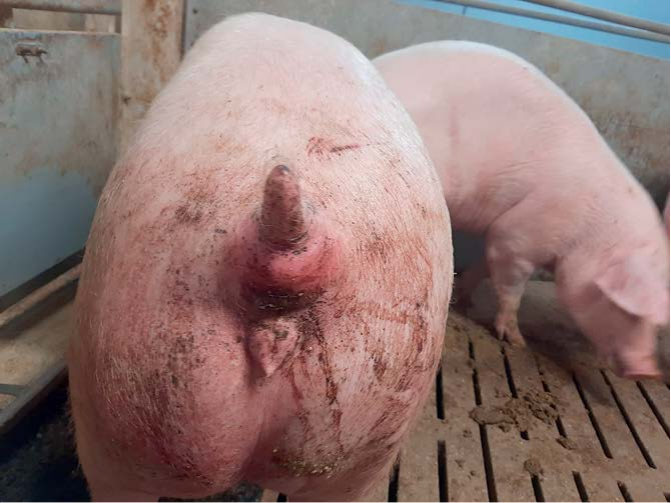
Umfangsvermehrungen zwischen Anus, Rektum und Schwanzansatz. Quelle: A. Schulte zu Sundern. **Fig. 1** Circumferential mass in the area ween the anus, rectum, and base of the tail. Source: A. Schulte zu Sundern.

Etwa 2 Wochen nach den reduzierten Futteraufnahmen traten an einzelnen Schweinen Umfangsvermehrungen zwischen Anus, Rectum und Schwanzansatz auf ( Abb. 1 ).

Im weiteren Verlauf vergrößerten sich diese Schwellungen und konnten bei immer mehr Tieren beobachtet werden. Letztendlich waren ca. 20 Endmastschweine betroffen. Die Ödeme im anorektalen Bereich eröffneten sich in den Folgetagen und wiesen einen eitrigen Inhalt auf. Daraufhin wurde der Tierarzt verständigt. Mögliche Ursachen wie mechanische Verletzungen und Kannibalismus waren auszuschließen, da sie nicht in einen kausalen Zusammenhang mit den beobachteten Veränderungen gebracht werden konnten. Die betroffenen Schweine waren in verschiedenen Stallabteilen und Buchten, klinisch allgemeingesund und nicht auffällig. Nach intensiver Recherche und einer ausführlichen Anamnese wurde vermutet, dass sowohl die Futterverweigerung als auch die eitrig-entzündlichen Veränderungen im anorektalen Bereich auf eine Kontamination des Futters mit Gewöhnlichem Stechapfel zurückzuführen sind. Die betroffenen Einzeltiere wurden antibiotisch und analgetisch behandelt. Die belastete CCM-Charge war zu diesem Zeitpunkt bereits vollständig gefüttert worden. Nach ungestörten Wundheilungen wurden die betroffenen Schweine am Mastende regulär geschlachtet. Weitere Fälle wurden nicht festgestellt.

## Diskussion

*Datura* sp. Ist ein unerwünschter Stoff in der Tierernährung und bei Kontamination von Mischfuttermitteln darf nach der Richtlinie 2002/32/EG der zulässige Höchstgehalt von 1000 mg/kg Mischfuttermittel (bei 88% Trockensubstanz) nicht überschritten werden. Im vorliegenden Fall konnte der genaue Anteil von Stechapfelsamen und Flüssigfutter in der betreffenden Charge retrospektiv nicht mehr bestimmt werden, weil der bestandsbetreuende Tierarzt zeitlich verzögert hinzugezogen wurde, d. h. als die betroffene CCM-Charge bereits vollständig an die Schweine gefüttert war. Der kontaminierte CCM wurde als Bestandteil einer Mischration eingesetzt. Im Anmischbehälter erfolgte unter Zugabe von Wasser eine Durchmischung der Futterkomponenten, wobei jedoch nicht ausgeschlossen werden kann, dass es auf dem Weg zum oder im Tog zu einer Entmischung kam. Dafür könnte sprechen, dass Stechapfelsamen auf der flüssigen Phase eines mit Wasser versetzten Mischfutters schwimmen, was eine Entmischung begünstigt. Es ist daher anzunehmen, dass die Aufnahme der toxischen Samenpartikel individuell unterschiedlich war. Die beobachtete Ausbildung von Abszessen könnte in Abhängigkeit von der aufgenommenen Dosis sowie individuellen Faktoren stehen, was erklären würde, warum klinische Veränderungen nur bei einzelnen Tieren manifest wurden. Da keine flächendeckende Untersuchung aller Tiere im Bestand durchgeführt wurde, kann jedoch nicht ausgeschlossen werden, dass weitere Tiere betroffen waren, insbesondere mit möglicherweise kleineren, weniger auffälligen Umfangsvermehrungen.

Mit den sich immer deutlich abzeichnenden Klimaveränderungen und dem weltweiten Handel mit Waren aller Art können sich immer mehr Neophyten ausbreiten und etablieren. Diese neuartigen Pflanzen haben zum Teil bei Kontakt oder Aufnahme unerwünschte Wirkungen. Bei der Produktion von heimischen Futter- und Lebensmitteln können diese bei Unkenntnis oder ungenügender Sorgfalt zu Problemen führen. Der Gemeine Stechapfel, *Datura stramonium* , gehört zur Klasse der Zweikeimblättrigen Pflanzen (Dicotyledoneae) und der Familie der Nachtschattengewächse. Die Pflanze stammt ursprünglich aus Amerika und ist einjährig 9 .

In allen Pflanzenteilen des Stechapfels befinden sich Tropanalkaloide. Die höchsten Konzentrationen lassen sich im Stängel (bis 9000 mg/kg TS) nachweisen. Die Samen enthalten im Vergleich dazu und zu den Blättern und Blüten geringere Konzentrationen 10 . Im hier beschriebenen Fall konnten ausschließlich Stechapfelsamen im Corn-Cob-Mix identifiziert werden. Andere Stechapfelbestandteile wie Stängel, Blüten und Blätter wurden wahrscheinlich im Ernteprozess ebenso wie die restlichen Pflanzenteile der Maispflanze zum allergrößten Teil aussortiert.

Eine Kontamination des Ernteguts durch austretenden tropanalkaloidhaltigen Pflanzensaft ist trotz eines hohen Trocknungsgrades des Stechapfels in den üblichen Erntemonaten (August bis Oktober) dennoch möglich 11 . Pflanzenbauversuche mit Weizen legen dieses nahe. Dabei reichten 1,5 kg Stechapfel (Frischmasse) auf einen ha schon für eine Überschreitung der geltenden Grenzwerte.

Gegen die Ausbreitung des Stechapfels im Bestand gibt es pflanzenbauliche Handlungsempfehlungen. Bei geringem Aufwuchs sollten zuerst immer mechanische Handlungen vorgenommen werden. Junge Pflanzen können relativ mühelos vor der Samenreife von Hand oder mit einer Hacke entfernt werden. Bei einer großflächigen Ausbreitung in der Kultur stößt dieses Vorgehen an seine Grenzen. In diesem Fall gibt es auch die Möglichkeit mit chemischen Mitteln eine weitere Ausbreitung und Kontamination der Feldfrüchte zu verhindern. Ferner konnten in Feldversuchen mit konkurrenzstarken Pflanzen eine Reduktion der Stechapfelbiomasse um ca. 50% erreicht werden 12 . Um eine Futtermittelkontamination mit tropanalkaloidhaltigen Pflanzenteilen des Stechapfels zu verhindern, sollte aber eine totale Elimination im Bestand erfolgen.

Dass das Füttern von Bestandteilen des Gewöhnlichen Stechapfels eine Wirkung auf das Hausschwein hat, ist schon lange bekannt. Während in frühen Veröffentlichungen wie „Die Krankheiten der Schweine“ aus dem Jahr 1842 noch eine narkotische Wirkung als förderlich für eine schnelle Mast angesehen wurde, stehen in späteren und neusten Publikationen die antinutritiven, d. h. unerwünschten, Effekte im Vordergrund. Als klinische Symptome, die bei Schweinen im Vergiftungsfall beobachtet werden, ist zuerst die Futterverweigerung zu nennen. Darüber hinaus konnten experimentell sowohl bei Schweinen, Ratten, Pferden und Meerschweinchen eine hemmende Wirkung auf die Darm- und Uterusmotorik ausgelöst werden 8 13 . Bei einer ausführlichen Allgemeinuntersuchung, die in der Nutztiermedizin weniger üblich ist und im dargebrachten Fall auch nicht erfolgte, können Symptome wie Mundtrockenheit, erhöhte Herzfrequenz und Pupillenweitung festgestellt werden 14 .

Während in der Veterinärmedizin bis jetzt anorektale Abszesse beim Schwein in Verbindung mit einer gehemmten Darmmotorik bzw. Verstopfung nicht in der wissenschaftlichen Literatur beschrieben wurden, ist in der Humanmedizin ein grundsätzlicher Zusammenhang zwischen Verstopfung und dem Vorkommen von anorektalen Abszessen aus der Beschreibung von einigen wenigen klinischen Fällen bekannt 15 . Dies aber jeweils ohne die Beteiligung von Stechapfel.

Im vorliegenden Fall, bei dem in ca. 20 Endmastschweinen anorektale Abszesse auftraten, kann über die genaue Entstehung nur gemutmaßt werden. Ähnliche, als Folge einer Aufnahme von Stechapfel entstandene Veränderungen wurden bis jetzt nicht bei Schweinen oder anderen Tieren beschrieben. In der Humanmedizin stellen anorektale Abszesse und Fisteln ein häufig auftretendes und gut erforschtes Feld dar 16 . Die Pathogenese der anorektalen Abszesse ist in den meisten Fällen kryptoglandulär. Ursächlich sind Infektionen der in der Darmwand befindlichen Krypten mit den dort mündenden Proktodealdrüsen. Es handelt sich dabei um merokrine Drüsen, die Muzin sezernieren. Durch abgestorbenes Gewebe oder Stuhl kann es zu einer Verlegung des Lumens der Proktodealdrüsen kommen. Daraus kann letztendlich eine Kryptitis oder eine sekundäre Abszedierung entstehen. Abszess und Fistel stellen dabei die akute und chronische Form des gleichen Krankheitsbildes dar. Bei der Fistel eröffnet sich der gebildete Abszess auf dem Weg des geringsten Wiederstandes in Richtung Hautoberfläche 17 . Im dargebrachten Fall konnte nicht festgestellt werden, ob die erkennbar eitrig-entzündlichen anorektalen Veränderungen eine Fistulierung in Richtung Darmlumen aufwiesen.

## Notes

Fazit für die Praxis Beim Einsatz von heimischen Futtermitteln sollte schon beim Anbau darauf geachtet werden, dass Nachtschattengewächse im Aufwuchs nicht dominant auftreten und nicht in relevanten Mengen gefüttert werden. Dem Tierhalter ist zu raten, sensorisch jedes Futtermittel zu überprüfen. Aus tierärztlicher Sicht ist es ratsam, bei totaler Futterverweigerung verdächtige Komponenten bis zur Klärung ihrer Identität bzw. möglichen Schadwirkung nicht zu füttern. Die im dargebrachten Fall beschriebenen anorektalen Abszesse sind bisher nicht in der veterinärmedizinischen Literatur beschrieben worden. Dass ein Zusammenhang mit der Aufnahme von Stechapfel und der damit möglicherweise verursachten Störung der Darmmotorik bestehen könnte, scheint soweit plausibel, da in der Humanmedizin zumindest der Zusammenhang von Verstopfung und der Entstehung von anorektalen Abszessen beschrieben worden ist. Wahrscheinlich hätten diese Abszesse durch einen Fütterungsstopp oder zumindest eine deutliche Reduktion der CCM-Komponente in der Ration verhindert werden können. Auch die Richtlinie 2002/32/EG bietet hier eine Orientierungshilfe für Höchstgehalte pflanzlicher Kontaminanten, sofern es sich wie beim Gewöhnlichen Stechapfel um einen unerwünschten Stoff im Sinne der Richtlinie handelt. In jedem Fall sollten betroffene Tiere tierärztlich untersucht und behandelt werden.
